# A Non-Synonymous Coding Variant (L616F) in the *TLR5* Gene Is Potentially Associated with Crohn's Disease and Influences Responses to Bacterial Flagellin

**DOI:** 10.1371/journal.pone.0061326

**Published:** 2013-04-11

**Authors:** Jared Sheridan, David R. Mack, Devendra K. Amre, David M. Israel, Artem Cherkasov, Huifang Li, Guy Grimard, Theodore S. Steiner

**Affiliations:** 1 Division of Infectious Diseases, Vancouver Hospital and University of British Columbia, Vancouver, British Columbia, Canada; 2 Division of Gastroenterology, Children's Hospital of Eastern Ontario, Ottawa, Canada; 3 Research Centre, CHU-Ste-Justine, Montreal, Canada; 4 Department of Pediatrics, University of Montreal, Montreal, Canada; 5 Division of Gastroenterology, Hepatology and Nutrition, British Columbia's Children's Hospital, Vancouver, Canada; 6 Prostate Centre, Vancouver Coastal Research Institute, Vancouver, British Columbia, Canada; Morehouse School of Medicine, United States of America

## Abstract

**Background and Objectives:**

Although numerous studies have implicated *TLR5*, or its ligands, bacterial flagellins, in the pathogenesis of Crohn's disease (CD), genome-wide association studies (GWAS) have not reported associations with the *TLR5* gene. We aimed to examine potential CD-associated *TLR5* variants and assess whether they modified inflammatory responses to bacterial flagellins.

**Methods and Principal Results:**

A two-stage study was carried out. In stage 1, we genotyped tagging single-nucleotide polymorphisms (tag-SNPs) in the *TLR5* gene in a sample of CD cases (<20 years of age, N = 566) and controls (N = 536). Single SNP and haplotype analysis was carried out. In Stage 2, we assessed the functional significance of potential CD-associated variant(s) vis-à-vis effects on the inflammatory response to bacterial flagellin using HEK293T cells. We observed marginal association between a non-synonymous coding SNP rs5744174 (p = 0.05) and CD. Associations between SNP rs851139 that is in high linkage disequilibrium (LD) with SNP rs5744174 were also suggested (p = 0.07). Haplotype analysis revealed that a 3 marker haplotype was significantly associated with CD (p = 0.01). Functional studies showed that the risk allele (616F) (corresponding to the C allele of SNP rs5744174) conferred significantly greater production of CCL20 in response to a range of flagellin doses than the comparator allele (616L).

**Conclusions:**

Our findings suggest that a non-synonymous coding variation in the *TLR5* gene may confer modest susceptibility for CD.

## Introduction

The toll-like receptors (TLR) are a group of receptors widely implicated in the regulation of innate responses in the intestine. They have been shown to contribute importantly to the pathogenesis of Crohn's disease (CD) via the identification of specific molecules on pathogens [1]. From among the various TLR's much interest is focused on TLR5 as this is the receptor that uniquely recognizes bacterial flagellin, a component of flagella and a highly prevalent antigen in the intestinal lumen. CD patients are significantly more likely than healthy controls or patients with ulcerative colitis (UC) to have serum antibodies to the CBir1, A4-Fla2, and related flagellins from gram-positive, anaerobic bacteria [2]. Further support for a potential role for TLR5 comes from recent observations that flagellins such as CBir1 are also dominant antigens in different models of experimental colitis [2]–[4].

In spite of the potentially important role of TLR5 in human CD, epidemiological studies investigating associations between genetic variants in the gene and CD have been equivocal. Barring one study that found negative associations between a *TLR5* nonsense mutation and CD in an Ashkenazi Jewish population [5], no other candidate gene or genome-wide association study (GWAS) has identified the *TLR5* gene as a susceptibility gene for CD. Given the need to stringently control for multiple comparisons in GWAS studies, associations with the *TLR5* gene may have been missed. As was shown for the IL10-gene [6]–[7] we speculated that if associations between the *TLR5* and CD exist, investigation in a pediatric cohort may provide additional insights. The major objective of the present study was thus to explore whether DNA variants across the *TLR5* gene were associated with CD in Canadian children and young adults and to examine whether associated variants if any, modulate inflammatory responses to bacterial flagellin.

## Methods

### Ethics statement

Ethical approval was acquired from the Ethics Review Board of the Ste-Justine Hospital Foundation (HSJ), Montreal; the Children's Hospital of Eastern Ontario (CHEO), Ottawa; and the British Columbia's Children's Hospital, Vancouver. Informed written consent was obtained from all participants (directly from the subject if he/she was an adult or from the parent/guardian if otherwise).

A case-control study was carried out at three pediatric gastroenterology clinics across Canada (Montreal, Ottawa & Vancouver). In brief, cases of CD were patients diagnosed using standard criteria prior to age 20 years. The phenotype of the cases at diagnosis was classified using the Montreal Classification. To ensure population representativeness, controls were selected from different sources that included children visiting the acute trauma clinics of the study hospitals, but who were otherwise healthy, their siblings, a birth cohort and population-based controls. Using these controls (and a subset of the cases) we have previously replicated associations with various CD-susceptibility genes [8]–[12]. Blood or saliva was collected from the participants as a source of DNA. Informed consent was acquired and the study was approved by the ethical review committees of the participating hospitals.

### Genotyping

The *TLR5* gene is located on chromosome 1 (1q41-1q42). It spans ∼33100 bp and demonstrates substantial variation. As many of these variations show high linkage disequilibrium (LD) we selected variants for genotyping using the tag-SNP method described by Carlson et al (2004) [13]. We restricted selection to variants with >10% frequency and LD to r^2^>0.8. Using this strategy we identified 4 variants for study that included the two common non-synonymous coding SNPs rs2072493 (A592S) and rs5744174 (F616L). An additional intronic SNP rs851139 that is in high LD with the coding SNP rs5744174 was also genotyped. Genotyping was carried out using the Sequenom platform (primers available on request) based at the McGill University & Genome Quebec Innovation Center in Montreal.

### Functional studies

Although none of the GWAS carried out either in adults or children demonstrated associations between the *TLR5* gene and CD at the genome-wide significance level, the WTCCC GWAS [14] demonstrated nominal associations (p<0.05) with two SNPs (rs851193, rs851192) in the gene. Both these SNPs are in perfect LD with the non-synonymous coding SNP rs5744174 that was included in our tag-SNP panel. Hence in parallel with the genetic association study we also carried out *in vitro* studies to assess the influence of this variant vis-à-vis its effects in determining inflammatory responses to flagellin.

A plasmid expressing the TLR5 616F allele under control of the EF-1α promoter (pEF6-V5His-TLR5) was obtained from Dr. Alan Aderem (Institute for Systems Biology, Seattle, WA). Quick-change mutagenesis was used to introduce the L allele, which was confirmed by PCR followed by sequencing of the plasmid.

HEK 293T cells were obtained from American Type Culture Collection (Manassas, VA) and grown in high-glucose DMEM (Invitrogen, Carlsbad, CA) with 10% heat-inactivated fetal bovine serum (Fisher, Ottawa, ON), penicillin, streptomycin, and 1× non-essential amino acids (Fisher). Cells were passaged twice weekly. For transfection experiments, they were seeded at 7×10^3^/well in poly L-lysine-coated 96-well plates in media without antibiotics, and transfected the following day using Lipofectamine 2000 (Invitrogen) according to the manufacturer's instructions. Cells were transfected with the following conditions, per well: pEGFP-N1 1 ng, and V5-TLR5616L 5 ng or V5-TLR5616F 2.5 ng, plus salmon sperm DNA to total 100 ng/well. Media was changed the following day, and cells were stimulated the day after that with recombinant *E. coli* H18 flagellin (FliC) prepared in *E. coli* BL21 as previously described [15]. After 3 h, human IL-8 and CCL20 concentrations in supernatants were determined by ELISA (OptEIA, BD Biosciences, San Jose, CA for IL-8 and Duo-set, R&D, Minneapolis, MN for CCL20) according to the manufacturers' instructions.

For reporter assays, cells were transfected as above, with the inclusion of p3X-NF-κBpLuc (obtained from S. Turvey, University of British Columbia) at 10 ng per well. Cells were stimulated for 6 h, and then frozen for subsequent luciferase quantification (BrightGlo, Promega, Madison, WI). Plates were read for fluorescence and luminescence, and the ratio taken in each individual well.

In each experiment, a subset of transfected but unstimulated wells were washed in cold PBS and lysed in 20 mM Tris pH 7.5, 150 mM NaCl, 1 mM EDTA, 1 mM EGTA, 1% NP-40, 2.5 mM sodium pyrophosphate, 1 mM β-glycerophosphate, 2 mM Na_3_VO_4_, and protease inhibitor cocktail (Sigma). Equal amounts of proteins were separated by SDS-PAGE and blotted for Western analysis. Blots were probed using anti-V5 (anti-PK, Serotec, Oxford, UK) and anti-GAPDH as a loading control. The density ratio of the V5 to GAPDH in each lane was calculated to verify equal expression of the two TLR5 constructs.

### Homology Modeling of Ectodomain of TLR5

As the ectodomain (ECD) of TLR5 is responsible for flagellin binding and the crystal structure of human TLR5 has not been obtained, homology modeling was used to generate a three-dimensional (3D) structure of TLR5 ECD. The ECD part (from 21–639) of the full-length sequence of the human TLR5 (Accession number: O60602) was used as the target sequence to do the blast, and 5 crystal structures of TLR3 and TLR4 (3CIG.pdb^1^, 2A0Z.pdb^2^, 1ZIW.pdb^3^, 3FXI.pdb^4^, 2Z64.pdb^5^) were ranked high based on the Max Score, Total Score and coverage. To get reasonable models, all five crystal structures were used as templates to provide more reference information for the sequence alignment. Multiple sequence alignment was performed to align the target sequence with template sequences within EasyModeller 2.1^6^. To compare the mutation of L to F at 616, TLR5 ECD with L616 (accession number: B9VJ73) was modeled based on the same templates. The stereochemical fitness of the two models was checked using Protein Geometry within MOE 2010 (Molecular Operating Environment).

Subsequent to this modeling, the crystal structure of zebrafish TLR5 [16] and the structure of the full-length human TLR5 by electron microscopy single-particle image reconstruction^18^ were published. Notably, these two models contain contradictory predictions about the structure of the flagellin/TLR5 complex. Both models were incorporated into the structure model generated based on TLR3 and TLR4 as above and did not change the results significantly.

### Statistical analysis

For genetic association analysis, each variant was assessed for departures from Hardy-Weinberg Equilibrium (HWE) using chi-square tests. The major allele of each SNP was considered the reference allele. Allelic associations were examined using logistic regression by assuming an additive model. This model is equivalent to the trend-test commonly utilized in GWAS. Haplotype analysis was carried out using HAPLOVIEW (www.broad.mit.edu/mpg/haploview) and observed p-values were corrected for multiple comparisons using permutation methods (n = 10,000).

For in vitro studies, chemokine or luciferase values were expressed as a ratio versus the highest value within each individual experiment (to correct for differences in absolute chemokine and luciferase expression between experiments due to slight variations in cell number or incubation time). The Y values were plotted against Log_2_ transformed FliC concentrations to generate a linear dose-response relationship for each TLR5 allele. Statistical testing by linear regression was performed using GraphPad Prism software to test for a difference between the dose-response curves.

## Results

### A non-synonymous coding *TLR5* SNP is associated with pediatric-onset CD

A total of 566 cases and 536 controls were studied. Table 1 shows the clinical and demographic features of the cases. The mean (SD) age at diagnosis was 12.3±3.3, most patients were male (57.8%), had ileo-colonic disease (48.8%) and inflammatory behavior (87%) at diagnosis.

**Table 1:** Clinical and demographic characteristics of the CD patients and controls.

Characteristic		Cases(N = 566)	Controls (N = 536)
Age at diagnosis (Mean (±SD))		12.3 (±3.3)	10.6 (±7.2)
Gender (%)	Females	239 (42.2)	198 (40.0)*
	Males	327 (57.8)	298 (60.0)
Ethnicity (%)	Caucasian	556 (98.2)	536 (100.0)
Disease location (%)^a^	L1±L4	124 (21.9)	
	L2±L4	162 (28.6)	
	L3±L4	276 (48.8)	
	Only L4	4 (0.7)	
Disease behaviour (%)^a^	B1±p	492 (87.0)	
	B2±p	37 (6.5)	
	B3±p	37 (6.5)	

* The gender of 40 controls (birth cohort) was not available.

a Disease location (L1 = isolated ileal; L2 = isolated colonic; L3 = ileo-colonic; L4 = upper tract) and behaviour (B1 = inflammatory; B2 = stricturing; B3 = penetrating; p =  perianal disease) was classified at diagnosis, according to WGO's Montreal classification.

**Figure 1:**
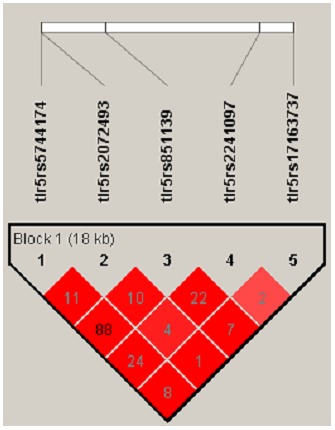
Linkage disequilibrium (r^2^) between the 5 SNPs in the *TLR5* gene.

All the genotyped SNPs were in HWE in the controls. Except for SNPs rs5744174 and rs851139 (r^2^ = 0.88) LD between the markers was low (Figure 1). Single SNP analysis (Table 2) showed that of the 5 SNPs examined, the non-synonymous coding SNP, rs5744174 (OR = 0.84, 95% CI = 0.71–1.00, p = 0.05) was significantly associated with CD. Given its high LD with rs5744174, there were also suggestions for associations with the intronic SNP rs851139 (OR = 0.85, 95% CI = 0.72–1.01, p = 0.07). No associations with the other non-synonymous coding SNP rs2072493 were evident. Haplotype analysis (Table 3) indicated that a high-frequency 3 marker (rs5744174, rs851139 and rs2072493) haplotype (TGA) was significantly (p = 0.01) associated with CD that persisted after corrections for multiple comparisons (permuted p-value = 0.04). The associations however disappeared after exclusion of the coding SNP rs5744174 from the haplotype.

**Table 2:** Association between SNPs across the *TLR5* gene and Crohn's disease.

SNP	Risk allele	Cases	Controls	Odds ratio	95% CI	P-value
rs5744174	C	451∶663	469∶583	0.84	0.71–1.00	0.05*
rs851139	A	419∶687	442∶618	0.85	0.72–1.01	0.07
rs2072493	G	160∶954	131∶919	1.18	0.92–1.51	0.20
rs2241097	C	273∶831	271∶777	0.94	0.77–1.14	0.55
rs17163737	A	120∶980	96∶962	1.23	0.92–1.63	0.16

* significant.

**Table 3:** Association between haplotypes comprising *TLR5* SNPs and Crohn's disease.

Haplotype	Case	Control	P Value
5 marker haplotype (rs5744174, rs2072493, rs851139, rs2241097, rs17163737)			
CAAAC	0.40	0.41	0.07
TAGCC	0.24	0.25	0.55
TGGAC	0.14	0.12	0.17
TAGAA	0.11	0.09	0.19
TAGAC	0.10	0.09	0.25
CAGAC	0.03	0.03	0.91
3 marker haplotype (rs5744174, rs851139 and rs2241097)			
CAA	0.38	0.41	0.07
TGA	0.35	0.30	0.01*
TGC	0.25	0.26	0.52
CGA	0.03	0.03	0.95

* significant.

### The CD-associated and comparator *TLR5* SNPs show functional differences

**Figure 2:**
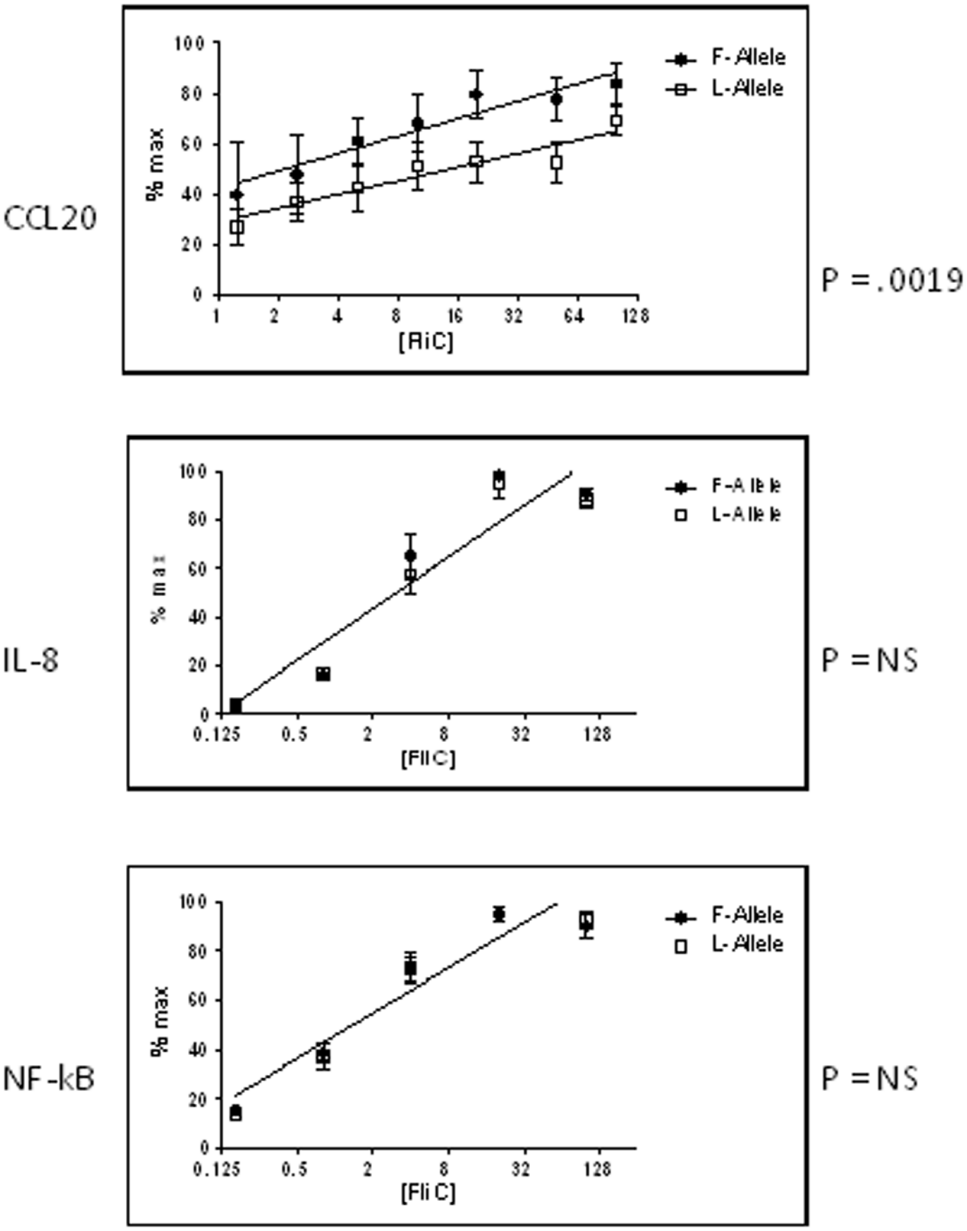
Functional differences between the 616F and 616L alleles of SNP rs5744174. HEK 293T cells were transiently transfected with NF-kBpLuc, pEGFP as a transfection control, and with plasmids expressing either V5-tagged TLR5 616L or 616F at amounts that generated equivalent expression of protein (verified by Western blot using anti-V5). Cells were stimulated with a range of flagellin doses for 6 h and supernatants collected for CCL20 (top) or IL-8 (middle) quantification. Cell lysates were analyzed for luciferase expression as a marker of NF-kB activity (bottom). Data were compiled from at least three independent experiments and normalized to the maximal value within each experiment. Sets were compared by linear regression of log-transformed data. The two lines were significantly different only for CCL20 expression, indicating a significantly greater CCL20 output with flagellin stimulation of the TLR5 616F allele.

To determine whether the risk allele of *TLR5* SNP rs5744174 has functional consequences, we used an HEK 293T flagellin response bioassay to compare the two variants of the SNP (616L and 616F). Proteins were transiently expressed in HEK cells stimulated with a range of flagellin doses, and three functional outputs were measured: NF-κB activation (via a luciferase reporter assay), release of IL-8 (CXCL8), and release of MIP3α (CCL20). As shown in Figure 2, there was no discernible difference between the two alleles in NF-κB activation or IL-8 expression. However, the risk allele (616F) conferred significantly greater production of CCL20 in response to a range of flagellin doses than the comparator allele (616L).

**Figure 3:**
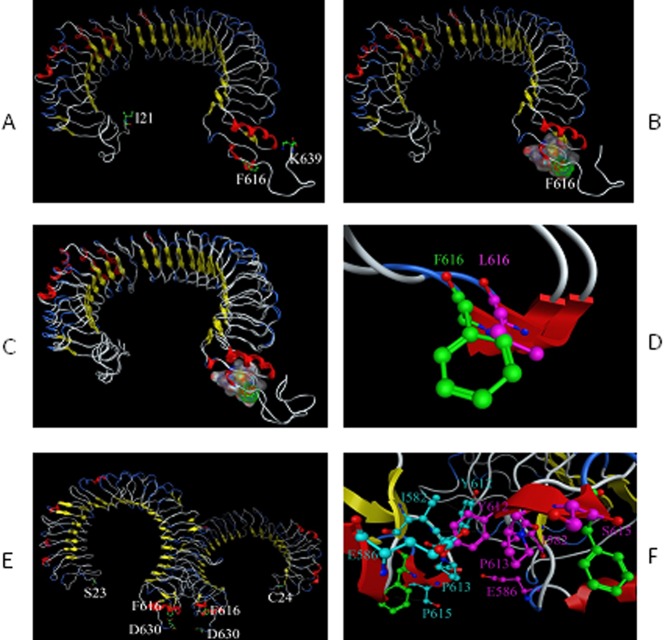
Structural correlates of the amino acid residue 615 on TLR5. Protein homology modeling of TLR5 suggests a possible contribution of the 616L/F residue to dimerization. (a) Representation of the 3D structure of TLR5 extracellular domain (ECD) model; (b) Surface of the residues around F616; (c) Superimposition of the two models of TLR5 ECD (F616, L616); (d) Superimposition of residues F616 and L616; (e) The hypothesized three dimensional structure of the TLR5 dimer; (f) Putative dimerization site residues including I582, E586, Y612, P613, and S615.

To determine the possible structural correlates of the 616 residue on TLR5, protein modeling was undertaken based on the partial crystal structure of TLR5 [16]–[17] and the full structures of TLR3 and TLR4 (Fig. 3). The 616 residue in human TLR5 is extracellular, lying between the leucine-rich-repeat (LRR) domains responsible for flagellin binding, and the single transmembrane domain. Of note, there is no known role for this region of TLR5. However, structural modeling suggests a previously unrecognized potential contribution to dimerization at this region of the protein, which could explain the effects of the 616 residue on signaling (Fig. 3).

## Discussion

We observed that a non-synonymous coding variant (rs5744174) in the *TLR5* gene was modestly associated with CD in children. Functional studies demonstrated that the risk (F) allele of the variant was associated with greater expression of the chemokine CCL20 in response to flagellin, although there was no difference in IL-8 expression or NF-κB activation. The role of TLR5 in intestinal inflammation and homeostasis remains somewhat unclear. A relatively common nonsense mutation in TLR5 that acts as a partial functional dominant negative was associated with protection against CD in one study [5] which would suggest that TLR5 activation drives inflammation in IBD. This would also explain the high prevalence of anti-flagellin antibodies in CD compared to healthy controls [2], [18]. However, the flagellins targeted by these antibodies are themselves poor TLR5 agonists, which calls into question the role of TLR5 in this phenomenon. Moreover, there is experimental evidence of a protective role of flagellin/TLR5 interaction in the gut. For example, *TLR5*-/- mice display a propensity to colitis [19]–[21]. Moreover, administration of flagellin, or an engineered TLR5 agonist based on the flagellin structure, was shown to protect against radiation-induced intestinal injury [22]–[24]. Hence, TLR5 likely plays a mixture of protective and harmful roles in intestinal homeostasis and disease.

The canonical TLR5 activation pathway involves MyD88-dependent phosphorylation of IRAK-1, leading to activation of intermediate signaling molecules that culminate in phosphor-activation of MAP kinases and IKK, ultimately resulting in NF-κB activation and a pro-inflammatory transcriptional program [25]. Both IL-8 and CCL20 have NF-κB response elements in their promoters, but there are significant differences in other transcription factor sites, which suggest that subtle differences in TLR5 signaling may have important functional outcomes. Indeed, the recently reported crystal structure of the zebrafish TLR5/flagellin dimer suggests that regions of flagellin required for TLR5 binding and for facilitation of TLR5 homodimerization are different [16]. Moreover, the inability to include the region corresponding to amino acid 616 of human TLR5 in the crystallized protein indicates that the true functional importance of this region is still uncertain. The uncertainty is enhanced by the data from the electron microsopy-based structure of full-length human TLR5, which predicted a different dimerization structure than the crystal structure data. Hence, functional analyses will ultimately be required to definitively appreciate the mechanisms of TLR5/flagellin dimerization.

Our results would indicate that on the whole, greater functional activity of the *TLR5* variant rs5744174 (as suggested by increased CCL20 expression in response to flagellin) is a risk factor for CD in children. Reasons for this remain speculative, but could include differences in recruitment of Th17 cells or dendritic cells (which express the receptor for CCL20, CCR6) to the intestinal lamina propria, leading to altered inflammatory tone. Our conclusions are limited by the need to use a non-intestinal cell line for functional studies, since human intestinal epithelial cells and leukocytes express native TLR5. Testing of a large cohort of volunteers with both SNPs of TLR5 at residue 616 would be required to draw conclusions about functional differences in flagellin response, because of naturally occurring polymorphisms in other inflammatory genes involved in chemokine and cytokine production. However, these future studies could provide interesting information to guide new diagnostic or therapeutic interventions for pediatric CD.
